# Susceptibility gene profiling elucidates the pathogenesis of inflammatory bowel disease and provides precision medicine

**DOI:** 10.1002/ctm2.1404

**Published:** 2023-09-01

**Authors:** Han Gao, Ruize Liu, Hailiang Huang, Zhanju Liu

**Affiliations:** ^1^ Department of Gastroenterology Center for Inflammatory Bowel Disease Research Shanghai Tenth People's Hospital of Tongji University Shanghai China; ^2^ Department of Medicine Analytic and Translational Genetics Unit Massachusetts General Hospital Boston Massachusetts USA

**Keywords:** Chinese population, East Asian, inflammatory bowel disease, personalized medicine, susceptibility gene

## 1 THE CHALLENGE OF GENOME‐WIDE ASSOCIATION STUDIES IN INFLAMMATORY BOWEL DISEASE

Inflammatory bowel diseases (IBD) are characterized by idiopathic chronic inflammation of the gastrointestinal tract, comprising the two major entities ulcerative colitis (UC) and Crohn's disease (CD). A series of studies have demonstrated that perturbations of the indigenous gut microbiota, persistent infections, environmental factors, and genetic variants contribute to dysregulated immune responses to microbiota, leading to the development of IBD.^1^, ^2^ The incidence and prevalence of IBD are increasing globally, particularly in developing countries. The precise etiologies and pathophysiology are unknown, and therefore medical therapy to cure these diseases is not yet available. Genome‐wide association studies (GWAS) have identified > 240 genetic loci associated with human IBD. However, most IBD genetic associations have been derived using individuals from European (EUR) ancestries, with only a few studies of much smaller sample sizes in East Asian (EAS) ancestries, particularly the Chinese population.^3^, ^4^

## 2 BREAKTHROUGH IN GENETIC CHARACTERISTICS OF IBD IN EAST ASIAN ANCESTRIES

**FIGURE 1:**
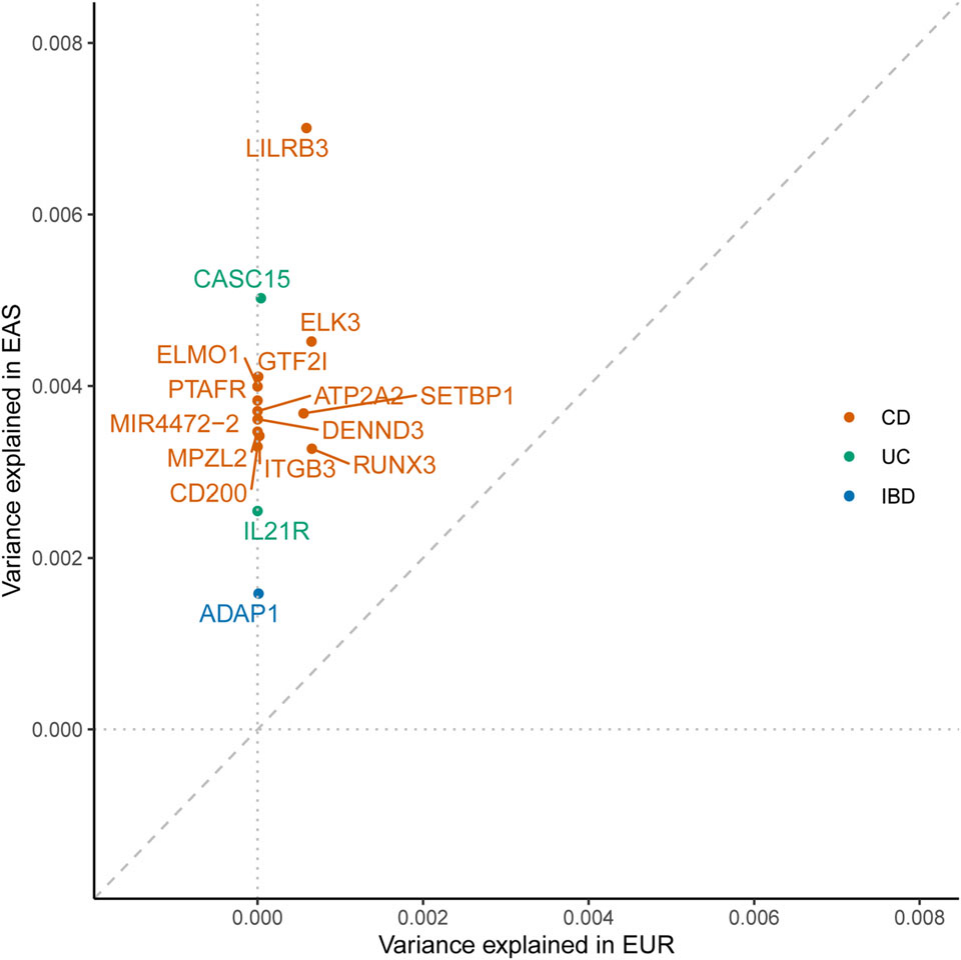
Variance explained for the 16 new IBD EAS loci across EUR and EAS. Index variants in the 16 new IBD EAS loci are plotted. IBD, inflammatory bowel disease; CD, Crohn's disease; UC, ulcerative colitis; EUR, European ancestry; EAS, East Asian ancestry.

Recently in *Nat Genet*, Liu et al. are the first to clarify the genetic architectures associated with human IBD using the largest samples from EAS ancestries including Chinese IBD patients.^5^ They identified 16 new genetic loci just across EAS ancestries. Variance explained for these IBD EAS loci across EUR and EAS was mainly by higher minor allele frequencies (MAFs) in EAS instead of similar effect sizes (average MAF 0.25 versus 0.17, two‐sided paired *t*‐test *p*  =  .015) (Figure 1), suggesting the importance of including global ancestries in genetic studies to ensure the relevance of the genetic findings.^5^ Furthermore, Liu et al. identified 81 new genetic loci associated with IBD across both EAS and EUR ancestries, thus increasing the number of IBD‐associated genetic loci to 320 by comparing with 45 106 IBD cases and 353 562 healthy donors from East Asian countries (China, Korea and Japan), the International IBD Genetics Union (IIBDGC) and FinnGen.^5^ Variance explained by IBD‐associated loci differs across EAS ancestries and EUR ancestries, which is, to a greater degree, driven by MAFs but less by the effect sizes (32% of IBD associations have different MAFs, and 22% have different odds ratios).^5^

## 3 GENETIC DIVERSITY OF POPULATION IMPROVES THE ABILITY TO PREDICT AN INDIVIDUAL'S DISEASE RISK OF IBD

Liu et al. have found that there is a comparable single nucleotide polymorphism‐based heritability across EAS ancestries and EUR ancestries, indicating that the amount of genetic contribution to IBD is roughly the same across the two populations. Besides, Liu et al. found that the genetic effects of many IBD loci are consistent in both ancestries, with only a few loci as clear exceptions, for example, tumour necrosis factor super family 15 (*TNFSF15*), colony‐stimulating factor 2 receptor subunit beta and interleukin 23 receptors. As the variance explained for IBD loci differs across ancestries, the ability to exploit genetic loci and predict an individual's disease risk of IBD also differs greatly. Liu et al. estimated this ability using polygenic risk score (PRS) and observed that PRS trained in the EUR population has a reduced accuracy in the EAS population. To some extent, the accuracy of predicting an individual's risk of CD or UC could be improved by jointly modeling EAS and EUR populations. Accordingly, the genetic diversity of the population plays a major role in GWAS for the equitable deployment of PRS in clinical settings.^5^

## 4 SUSCEPTIBILITY GENE PROFILING BENEFITS TO PRECISION AND PERSONALIZED MEDICINE

Susceptibility genes are significantly related to clinical manifestations, course of disease, and treatment response. Previous studies have shown that variants in nudix hydrolase 15 are associated with an increased risk of thiopurine‐induced myelosuppression and that these variants have a great predictive power for thiopurine‐induced myelosuppression.^6^ Precision and personalized medicine based on individual pharmacogenomic information enable the safe use of thiopurines by minimizing thiopurine‐induced severe side effects (e.g. leukopenia and alopecia) in IBD patients. Moreover, the *ATG16L1* genotype in CD patients largely affects the efficacy of corticosteroids, immunosuppressants, and biological agents.^7^ As a canonical autophagy‐regulating protein, ATG16L1 has been found to be downregulated in different types of tissues in Chinese CD patients, suggesting that a conjoint analysis of protein and gene enables us to screen more variants in different populations.^8^ Previous reports have shown that the *IL‐23R* genotype is an optimal predictor of an early response to anti‐TNF‐α monoclonal antibody (i.e., infliximab) in UC patients.^9^ Accumulating studies have tried to explore the association between the genetic characteristics and clinical phenotypes and outcome and found that nucleotide‐binding oligomerization domain containing 2 (*NOD2*) gene polymorphisms are associated with age and complications of CD patients. Importantly, the most common genetic variant of *ATG16L1* (rs2241880) is significantly associated with the development of CD perianal disease.^7^

## 5 FUTURE OUTLOOK

**FIGURE 2:**
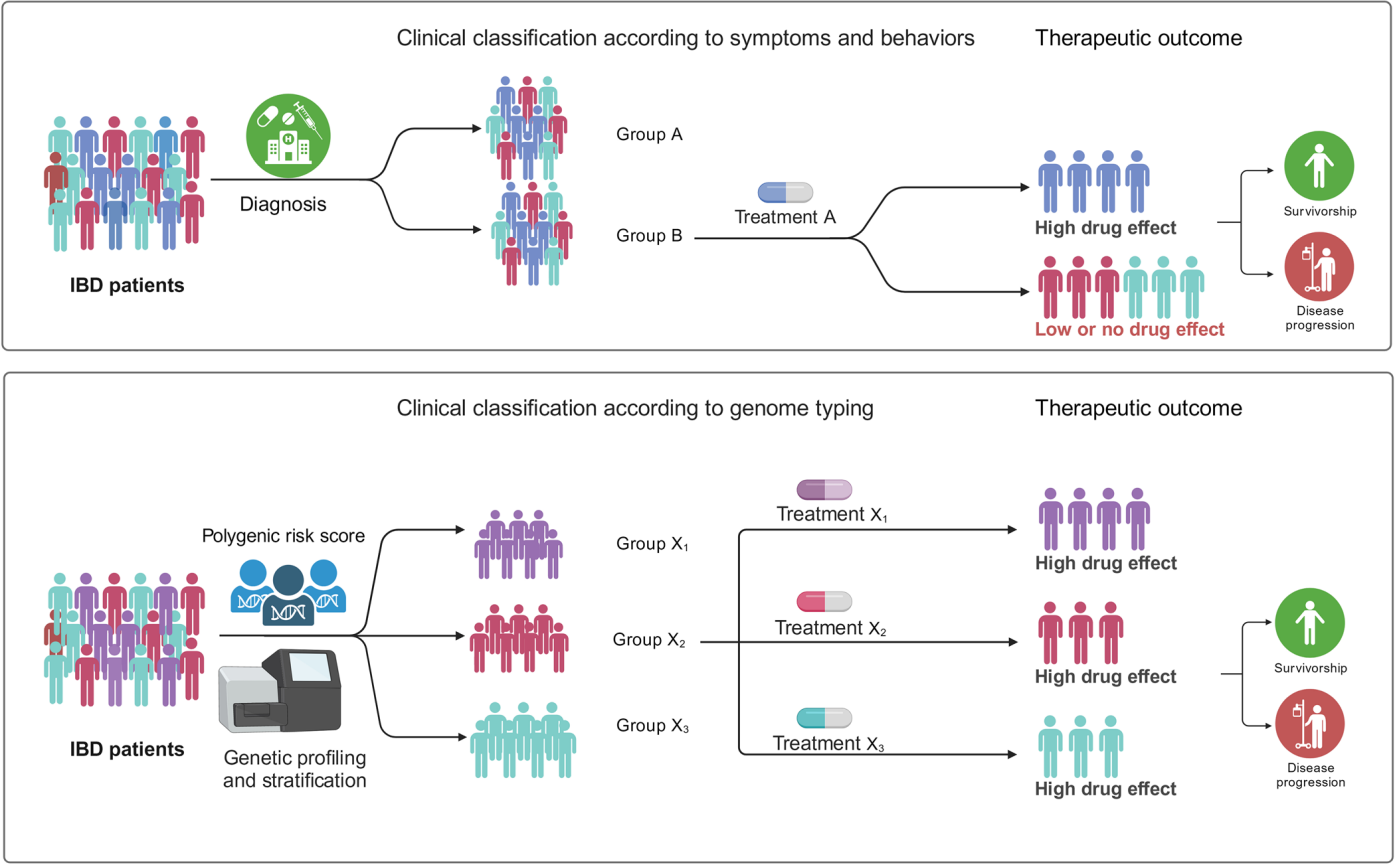
Susceptibility gene profiling benefits to precision and personalized medicine.

Precision and personalized medicine is a hotspot of IBD study. The data converging multiple omics, data integration, computer networking models, and new clinical trials lay the foundation for precision medicine in the future.^10^ Liu et al. have highlighted the similarities and differences in the genetic architectures between EUR and EAS ancestries and pinpointed the clinical significances relevant to IBD genetic study for the first time.^5^ The latest research progress in genetic characteristics of IBD in Chinese and other EAS ancestries will help us to better understand the pathogenesis of IBD and provide precision medicine in these emerging regions with high incidences of disease, particularly in EAS countries (Figure 2).

## CONFLICT OF INTEREST STATEMENT

The authors declare no conflict of interest.
